# *When the Renal (Function) Begins to Fall*: A Mini-Review of Acute Kidney Injury Related to Acute Respiratory Distress Syndrome in Critically Ill Patients

**DOI:** 10.3389/fneph.2022.877529

**Published:** 2022-04-21

**Authors:** Antoine Marchiset, Matthieu Jamme

**Affiliations:** ^1^ Service de Médecine Intensive Réanimation, Centre Hospitalier de Poissy-Saint Germain en Laye, Poissy, France; ^2^ Service de Réanimation, Hôpital Privé de l’Ouest Parisien, Ramsay Générale de Santé, Trappes, France; ^3^ INSERM UMR 1018, Equipe Epidémiologie Clinique, CESP, Villejuif, France

**Keywords:** acute kidney injury, acute respiratory distress syndrom (ARDS), lung kidney interaction, mechanical ventilation, COVID

## Abstract

Acute kidney injury (AKI) is one of the most frequent causes of organ failure encountered in patients in the intensive care unit (ICU). Because of its predisposition to occur in the most critically ill patients, it is not surprising to observe a high frequency of AKI in patients with acute respiratory distress syndrome (ARDS). However, few studies have been carried out to assess the epidemiology of AKI in subgroups of ARDS patients using recommended KDIGO criteria. Moreover, the mechanisms involved in the physio-pathogenesis of AKI are still poorly understood, in particular the impact of mechanical ventilation on the kidneys. We carried out a review of the literature, focusing on the epidemiology and physiopathology of AKI in patients with ARDS admitted to the ICU. We addressed the importance of clinical management, focusing on mechanical ventilation for improving outcomes, on AKI. Finally, we also propose candidate treatment strategies and management perspectives. Our literature search showed that AKI is particularly common in ICU patients with ARDS. In association with the classic risk factors for AKI, such as comorbidities and iatrogeny, changes in mechanical ventilation parameters, which have been exclusively evaluated for their outcomes on respiratory function and death, must be considered carefully in terms of their impact on the short-term renal prognosis.

## Introduction

Acute kidney injury (AKI) is the most common type of organ failure observed in patients in the intensive care unit (ICU) observed in its most severe form in about one-third of ICU patients (1). Surprisingly, few studies have assessed the relation between acute respiratory distress syndrome and AKI, and then only in specific contexts such as trauma (2), influenza (3), after lung transplantation (4), or intoxication (5). In an observational study specifically studied AKI in ARDS patients, 1879 patients were compared with 6150 patients without ARDS. The authors reported that 44% of ARDS patients had AKI and half of these had the most severe form. When compared to non-ARDS patients, this represented a 1.6-fold increase of incidence of AKI, with a strong statistical association between ventilation for ARDS and the occurrence of AKI after adjustment (OR=11[6.8-17.7]) (6). Using the most recent definition of AKI reported in the Kidney Disease Improving Global Outcome (KDIGO) guidelines, Panitchote et al. performed a competing risk survival analysis in a monocentric cohort of 634 ARDS patients. They observed AKI in more than two-thirds of patients and identified severe AKI in 118 (48%). Since December 2019, ICUs around the world have faced a significant increase in admissions for a particular form of ARDS, namely severe COVID-19. Marked by a particularly high rate of AKI [with a reported incidence of >80% in ICU patients receiving mechanical ventilation (MV)] (7) during the first wave in each country, a recent large observational database analysis from the UK observed a decrease in rate of AKI during successive waves in patients hospitalized with COVID-19 (8). Thus, the actual incidence of severe AKI appears to be around 25% and not to be greater than that in ARDS patients without COVID-19 (9). While AKI is associated with poor outcomes in all ICU patients (10, 11), the occurrence of renal failure during ARDS management appears to be a specific unfavorable risk factor for nosocomial pneumonia (12), an event leading to extended ventilation times (13), and for a lower rate of successful weaning from MV (14), which is responsible for an increase in overall mortality (10, 11),

The aim of this mini-review is to outline our current knowledge on the pathophysiological mechanisms involved in the development of AKI during the management of ARDS in ICU patients and to propose therapeutic approaches to limit its consequences.

## Methods

We conducted a literature search of available sources describing AKI in patients with ARDS. Research articles were selected on the basis of research topics found in the Web of Science, PubMed, Springer, and Scopus databases. These articles were classified according to their relevancy. The information found in the selected articles was evaluated and is described and discussed in the following sections.

## Existing Pathophysiological Models

### Patients With ARDS Have a High Baseline Risk of AKI

**Figure 1:**
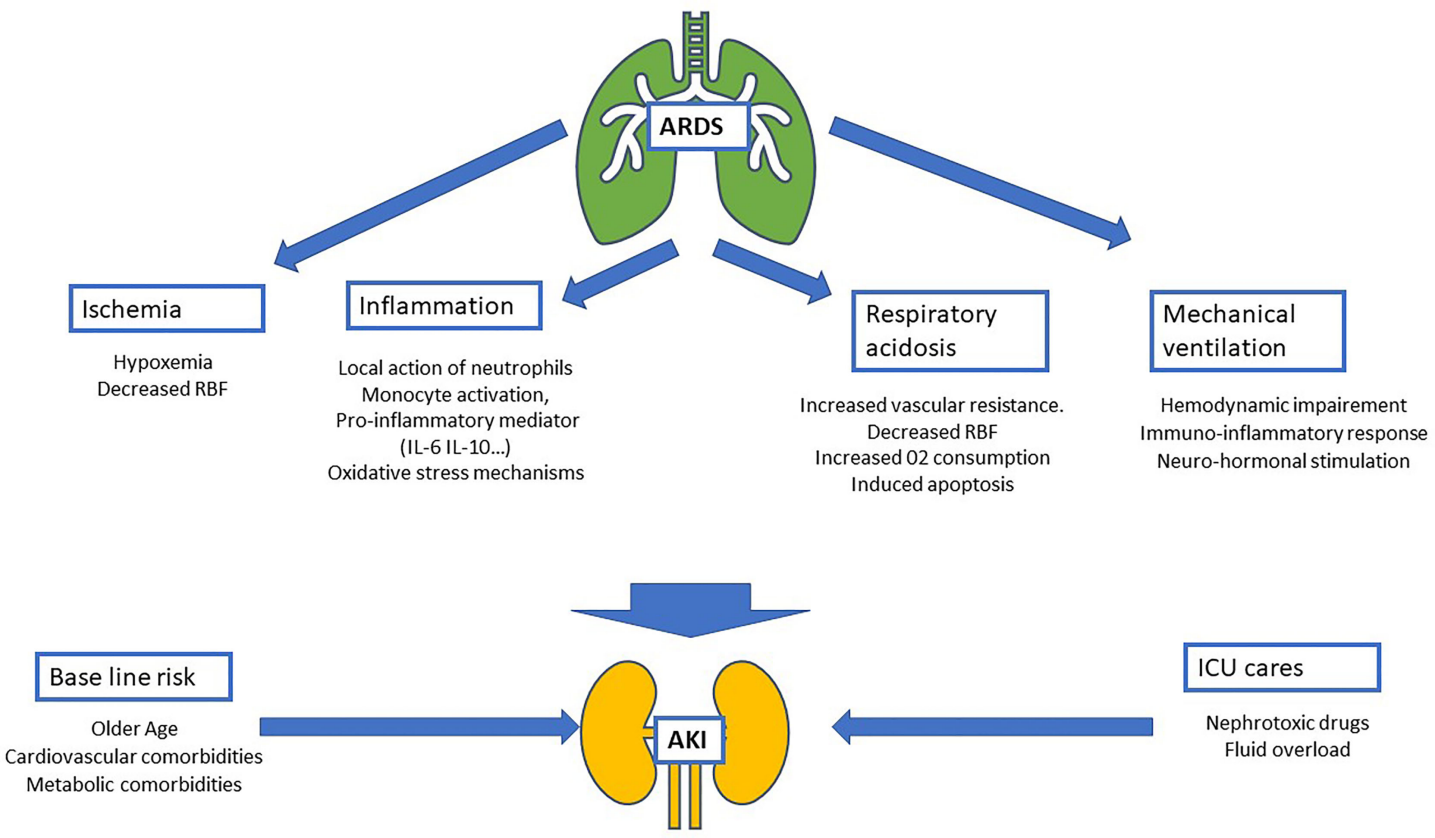
Schematic of physiopathology of acute kidney injury (AKI) during acute respiratory distress syndrome (ARDS). Four mechanisms related to ARDS are identified: ischemia, inflammatory, respiratory acidosis and the impact of mechanical ventilation. These mechanisms added to AKI baseline risk (mostly composed by comorbidities and age) and intensive care unit nephrotoxic treatment and procedures (nephrotoxic drugs, iodinated contrast products…). RBF, renal blood flow; IL-6, interleukin-6; IL-10, interleukin-10.

Epidemiological studies focusing on AKI have highlighted several baseline characteristics as risk factors for renal failure (1, 15). Overall, patients with AKI are frequently male, older, with a history of comorbidities such as arterial hypertension, diabetes, and cardiovascular diseases (16). These characteristics are common to those observed in patients admitted to the ICU for ARDS in the LUNG-SAFE study. Taken together, these data give patients admitted to the ICU with ARDS a high-risk of renal events (**Figure 1**).

#### The “Usual Suspects” for ARDS-Associated AKI: Ischemia, Inflammation, MV, and Respiratory Acidosis

##### Ischemia

In kidney tissue, tubular proximal cells, which are metabolically hyperactive cells with a high demand for oxygen to reabsorb water and electrolytes, must operate in a low oxygen environment because of the cortex-to-inner oxygen gradient. During ARDS, renal ischemia results principally from hypoxia, defined by an arterial blood oxygen level <75 mmHg. A decrease in oxygen results in a mismatch between local tissue oxygen supply and demand, and the accumulation of metabolic waste products. As a consequence, tubular epithelial cells undergo injury and death by apoptosis and necrosis with functional impairment of the kidney (17).

In addition to this direct effect due to the decrease in blood oxygen levels, ARDS, when associated with shock, could decrease renal blood flow (RBF) leading to renal ischemia by hypoperfusion. Moreover, hypoxemia seems to have a synergistic effect on RBF and is associated with a greater change in renal function (18) (**Figure 1**).

##### Systemic Inflammation

It has been suggested that some particular etiologies of ARDS are associated with a higher incidence of AKI. More frequently observed in sepsis (19), and/or in acute pancreatitis (20), and/or burns, and less frequently observed in toxic inhalation and/or viral pneumonia (21, 22), this reinforces the hypothesis that inflammation causes renal injury.

Inflammation has been already identified as one of the major mechanisms leading to the development of AKI, particularly during sepsis with: (i) the local action of neutrophils (PNN) through the secretion of pro-inflammatory cytokines including interleukin (IL)-1β and tumor necrosis factor (TNF)-α (23); (ii) monocyte activation, both pro- and anti-inflammatory, responsible for monocyte infiltration guided by chemokine receptor expression in the kidney (24); and (iii) the impact of a diffuse inflammatory state with significant renal toxicity of the immune response, in particular innate immunity (25).

Two pro-inflammatory mediators, IL-6 and IL-10, have been specifically identified as being involved in the development of renal lesions in murine models of sepsis (26, 27). The link between high IL-6 and IL-10 plasma concentrations and the development of AKI has also been confirmed in humans (25). Other inflammatory biomarkers also seem to participate in the development of renal dysfunction, such as TNF-21 (28), IL-8 (29), and vascular endothelial growth factor (VEGF) hypersecretion, *via* oxidative stress mechanisms (30).

In ARDS, lung injury is responsible for the release of pro-inflammatory mediators similar to sepsis (IL-6, PAI-1, soluble TNF receptors) (11). Systemic release of these inflammatory mediators has been identified as a pathway involved in AKI development during infectious lung injury (11, 31) (**Figure 1**).

##### Mechanical Ventilation

In addition to the impact of MV on pulmonary tissue, positive pressure ventilation also seems to have an impact on the kidney. Originally described in 1947 by the demonstration of a decrease in urinary flow in healthy volunteers (32), several fundamental studies have suggested the involvement of hemodynamic (33, 34), immuno-inflammatory (35–37), and neuro-hormonal (38–40) mechanisms that may alter renal function after the MV initiation.

The hemodynamic impact of MV results from interactions between the heart, kidneys, and lungs. The introduction of positive-end expiratory pressure (PEEP) by MV is not without consequences. The increase in intrathoracic pressure leads to a decrease in heart venous return and, as a consequence, a decrease in cardiac output (41–43). More recently, in a large cohort of ICU patients, a strong link was found between the PEEP level administered through MV and AKI in association with higher venous congestion pressure. The authors hypothesized that high PEEP could disturb renal perfusion *via* mechanisms linked to an alteration in mean arterial pressure and by an increase in induced venous congestion (44). In addition to the hemodynamic impact, MV was suspected to stimulate the renin angiotensin aldosterone system (RAAS), with a PEEP level-dependent effect (38). In addition to RASS, other hormonal responses are also altered during MV, especially the secretion of antidiuretic hormone and type A natriuretic peptide, without any formal link being established between these responses and the development of AKI (38, 45, 46).

Added to the effects of pulmonary damage related to ARDS, MV also induces pro-inflammatory mechanisms that have a direct impact on renal tissue through neutrophil activation in the intrapulmonary circulation (47) and/or pro-inflammatory cytokine release in animal models (48). In humans, the interaction with initial inflammation associated with ARDS makes it difficult to extrapolate these results, but several pro-inflammatory mediators seem to be increased by MV (TNF-α, IL-6, IL-8, PAI-1, TNF receptor(R)-1, and TNFR) (11, 31, 49) (**Figure 1**).

### Respiratory Acidosis

Over the past 10 years, a consensus has emerged concerning the need to limit plateau pressure during MV while maintaining a lung recruitment strategy with limitation of tidal volume (50). The so-called permissive hypercapnic acidosis observed at the “price” of this strategy has even been suggested to be one of the benefits of lung-protective ventilation (51). However, this point of view was challenged by several studies which suggested that hypercapnia may impair renal hemodynamics. In particular, it has been found that hypercapnia may induce vasoconstriction of the renal vascular bed leading to increased vascular resistance (52–54). A pCO_2_ threshold of 50 mmHg has even been identified as being responsible for the decrease in RBF (53, 55). In terms of cellular metabolism, respiratory acidosis may induce an increase in oxygen consumption in the proximal tubule (54, 56, 57). Furthermore, the hypercapnia/hypoxia combination has been shown to be synergistic in decreasing RBF and the development of ischemic apoptosis in renal tubule cells (58–61) (**Figure 1**).

### The Special Case of Severe COVID-19

The relationship between renal and pulmonary involvement in a specific model of ARDS has been questioned in the context of the current SARS-COV-2 pandemic. Although renal damage is the second most frequent type of organ damage during COVID-19 (62), a histopathological study showed that acute tubular necrosis was the main lesion observed in ICU patients, without viral material identified in kidney lesions (63), suggesting a predominant ischemic process for AKI. Recently, Sullivan et al. reported a dramatic decrease in rates of AKI after the first wave of COVID-19, and a lower rate of renal failure in older patients (8). Because of recommendations early during the pandemic, in particular warnings of the potential risk of aerosol formation that could increase the risk of contamination for healthcare workers, non-invasive ventilation (NIV) and high-flow nasal cannula oxygen (HFNO) therapy were used in only a minority of patients during the first wave (6). It was later shown that the risk of aerosolization with HFNO and NIV was similar to that with standard oxygen therapy and HFNO was suggested as first-line ventilatory support in COVID-19 patients with acute respiratory failure during subsequent waves of the pandemic (7). The lower rate of AKI observed after the first wave and in older patients, in whom the use of invasive ventilatory support was probably low, suggests the impact of MV on the development of AKI. These results are in line with those reporting MV as a major risk factor for AKI in patients with severe COVID-19 (7, 9, 63).

### The Usual Culprit, Septic Shock

Septic shock is frequently complicated by ARDS and is also a risk factor for AKI. Bellani et al. found an incidence of extrapulmonary septic shock in ICU patients with ARDS of 17.3% (16). The diffuse inflammation resulting from sepsis is responsible for a renal toxicity of the immune response (25), *via* pro-inflammatory cytokines (IL-6, IL-10, IL-1β, TNF-α) (23) and a monocytic infiltration of renal parenchym (24). Furthermore, hemodynamic failure in sepsis is linked to the occurrence of AKI (64). The SEPSIS-PAM trial confirms the importance of the link between blood pressure and AKI in sepsis (65). Vasopressors can improve filtration fraction, and normalize renal hemodynamic (66) but sympathetic overstimulation with high doses of catecholamines could be harmful (67).

## Managing AKI in Patients With ARDS

### “AKI Is Coming”

The cornerstone of AKI prevention in ICU patients is the management of hemodynamics, including the use of appropriate volumes of fluids, the choice of fluids, and the choice of vasoactive drugs. Although the pathogenesis of AKI in ARDS patients depends on different mechanisms, hemodynamic optimization appears to be essential to prevent alterations in RBF (**Table 1**).

**Table 1:** Therapeutic measures proposed before and during and after AKI during ARDS.

	Effective	Not proven effective	Still on debate
**Preventive measures to limit AKI occurrence**			
Risk stratification by AKI risk model in context of specific AKI (nephrotoxic drugs, post surgery)	X		
Risk stratification by AKI risk model concerned all patients admitted in ICU			X
Risk stratification using biomarkers of renal aggression			X
Avoid hypovolemia condition	X		
Avoid use of synthetic colloïds for restore hypovolemic condition	X		
Avoid fluid overload	X		
Target a threshold of 85mmHg of MAP in patient with history of arterial hypertension			X
Using vasopressin as first choice of vasopressors		X	
Control of blood glucose concentration			X
Use erythropoeitin to limit damage of renal ischemia		X	
Use steroïds to limit sepsis related AKI		X	
Monitoring PEEP according right ventricular function			X
Avoid severe hypercapnia			X
Adopt bundle of care specific to AKI	X		
**Therapeutic measures of AKI**			
Promoting use of cyrstalloïds for volume replacement	X		
Using vasodilatators agents in order to increase renal perfusion		X	
Research and consider to stop all drugs suspected as nephrotoxic	X		
Restore PGC1α-NAD pathway by B3 vitamin supplementation			X
Not starting too early RRT	X		
Promoting use of convective renal replacement methods to limit renal aggression		X	

AKI, acute kidney injury; ARDS, acute respiratory distress syndrome; MAP, mean arterial pressure; PEEP, positive expiratory end pressure; PGC1α-NAD, PPAR gamma coactivator 1 alpha nicotinamide adenine dinucleotide.

### Hemodynamic Management

Appropriate volume replacement should be performed as early as possible, bearing in mind that fluid overload is reported to be associated with a poor prognosis in ARDS patients (68). Crystalloids are the solutions of choice for ICU patients (69, 70). Because of the supra-physiological concentration of chloride (154 mmol/L; representing 1.5-times normal serum chloride concentrations), 0.9% saline solution had been incriminated in the development of hyperchloremia acidosis in patients receiving high volumes of solution. The results from a recent meta-analysis of 13 trials comprising more than 35000 patients suggested a better global and renal outcome with a balanced solution compared to 0.9% saline solution (OR of major adverse kidney events occurring in the first 30 days=0.78[0.66-0.91]; *I* ^2^ = 42%) (71).

Further to the choice of solute, the concept of target optimal mean arterial pressure (mABP) has long been advocated. In the EPI-AKI study, the factors associated with AKI included a past medical history of hypertension or shock at ICU admission, with a higher simplified acute physiology score 3 (1). This relationship was demonstrated in physiological studies, which strongly suggested that glomerular filtration rate and RBF can vary widely across mABP and the impact of increasing mABP on renal hemodynamics varies on an individual basis (72) (**Table 1**).

### Improve the Balance Between Oxygen Supply/Needs

Numerous other procedures aimed at improving intra-renal perfusion or oxygenation have been evaluated, including renal vasodilators, control of renal hypercatabolism, anti-inflammatory and antioxidants drugs. Among them, dopamine is undoubtedly the most extensively studied. Its administration at low doses induced a dopaminergic and β-adrenergic effect, resulting in renal vasodilation. However, despite intensive research, the data on preventing the development of AKI remain largely inconclusive (73). Other vasodilators have failed to show any renal benefit (74–77). Erythropoietin, steroids, tight glucose control, and numerous metabolic interventions have also been evaluated for the prevention of kidney damage in various conditions. Except for the control of blood glucose level, for which conflicting results have been obtained (78, 79), no renal benefit has been observed with any of these metabolic interventions (80–82) (**Table 1**).

### Kidney-Lung Protective Ventilation

In the more specific context of ARDS, optimization of ventilation parameters to limit the impact of MV on renal hemodynamics as much as possible, through venous congestion or right-sided heart failure, seem to be an interesting approach to prevent this serious complication, within the limits allowed by the patient’s respiratory severity and oxygenation needs (83). Right ventricular (RV) function is frequently altered in ARDS patients with an incidence of acute cor pulmonale of 25%, resulting in detrimental hemodynamic consequences (84). The choice of PEEP strategy is still under debate. While some authors have observed a respiratory improvement with high level PEEP strategy (85), others have reported that aiming for higher levels of PEEP worsened RV systolic function impairment and a lower PEEP application reduced RV dysfunction, suggesting an association with lower pulmonary vascular resistance (86). In a prospective physiological study, Mekontso Dessap et al. showed that increasing PEEP strategies at constant Pplat during severe ARDS induced marked hypercapnia, lower pH values and was associated with impaired RV function (83). These results suggest that lung protective ventilation should be adopted gradually to limit hypercapnia to improve RV function and renal hemodynamics in at-risk patients (**Table 1**).

### Bundles

Further to a single intervention, “bundles” have been proposed to prevent AKI (87, 88). Bundles are a small, straightforward set of evidence-based practices that have been proven to improve patient outcomes when performed collectively and reliably. This seems to allow better recognition (89) and reduce the risk of AKI progression (90). The implementation of bundles has been shown to result in a lower incidence of AKI in specific settings, such as nephrotoxic AKI or post-cardiac surgery (91, 92). However, it is unknown whether implementing these bundles in general ICU populations or in patients with sepsis could prevent AKI (**Table 1**).

## During AKI: Limiting the Severity and Improving Early Recovery From AKI

### Earlier Detection of Renal Damage

Screening, mainly by identifying pre-existing co-morbidities, will help to identify patients who are at risk of renal damage. Early detection of AKI is also necessary so that it can be managed more effectively. Since creatinine levels can increase late in the course of kidney damage, with thresholds being exceeded only after a 50% loss of renal function, the use of more sensitive markers to detect subclinical AKI is required. Early markers of tubular damage (tissue inhibitor of metalloproteinase 2, insulin-like growth factor-binding protein 7, kidney injury molecule-1, neutrophil gelatinase-associated lipocalin, IL-18) have been identified but are not yet available routinely (93). Biomarkers are supplemented by radiological examination with contrast ultrasound techniques to improve the assessment of lesion severity and recovery potential (94).

### Activation of the PGC1α-NAD Pathway

While no specific treatment for AKI is currently available, numerous advances in our understanding of the mechanisms leading to AKI in ischemic conditions have been made over the past few years. Among them, the PPAR gamma coactivator 1α nicotinamide adenine dinucleotide (PGC1α-NAD) pathway is one of the most promising targets to prevent AKI. As renal proximal tubular cells are one of the most energy or ATP demanding cells in the body, they are very dependent on mitochondrial function. An increase in the expression of PGC1α in renal epithelial cells subjected to ischemic stress was found to be protective, with an increase in NAD+ (95, 96). Furthermore, decreased expression of PGC1α was observed in kidney biopsies from patients with AKI (96). PPAR agonists have been proposed to prevent AKI induced ischemia-reperfusion (97). Nicotinamide, the amine form of vitamin B3 (niacin), was identified as a potential stimulator of NAD+ production (98). After promising preclinical experiments, nicotinamide administration was evaluated for the prevention of postoperative AKI after in a single center trial with encouraging results (99). In this phase 1 pilot study, 37 patients who had undergone cardiac surgery were randomly assigned to one of three groups: placebo, nicotinamide 1g/day, and nicotinamide 3g/day. The areas under the curve of all longitudinal serum creatinine levels measured after randomization were higher in the placebo group compared to patients who received nicotinamide supplementation. While these results deserve to be reassessed in larger populations with a more suitable outcome, emerging data linking the NAD+ equilibrium to AKI resistance opens a new exciting chapter in AKI research (98, 100).

### Renal Replacement Therapy: At the Right Time for the Right Patient

Over the past few years, the timing of renal replacement therapy has received particular attention in critically ill patients (101–105). ARDS patients were studied specifically in a *post-hoc* analysis of an AKI study, but no significant impact of early renal replacement therapy on 60-day mortality was observed (101). Concerning renal recovery, although higher renal replacement therapy-dependence among survivors at day-90 was observed in the STARRT-AKI study (85/814 (10.4%) for the accelerated group vs. 49/815 (6.0%) for the standard group), these data were observed in unselected ICU patients and are difficult to extrapolate to the ARDS population.

## Conclusion

AKI is a frequent complication in ARDS patients and has a significant impact on outcome. MV may favor the emergence of this complication. Although the prevention of renal failure remains difficult, the early identification of high-risk patients, the use of bundles, and personalized ventilation should be an integral part of the management of ARDS patients, in the absence of any new therapeutic targets.

## Author Contributions

AM performed review of the literature and designed figure. AM and MJ wrote the manuscript. All authors read and approved the final manuscript. The authors received no financial support for the research, authorship, and publication of this article.

## Conflict of Interest

MJ reports lectures fees from Alexion.

The remaining author declares that the research was conducted in the absence of any commercial or financial relationships that could be construed as a potential conflict of interest.

## Publisher’s Note

All claims expressed in this article are solely those of the authors and do not necessarily represent those of their affiliated organizations, or those of the publisher, the editors and the reviewers. Any product that may be evaluated in this article, or claim that may be made by its manufacturer, is not guaranteed or endorsed by the publisher.
